# Recrystallization Behavior in the Notch Region of AA8014 Aluminum Alloy Burst Vents and Its Effect on Burst Pressure

**DOI:** 10.3390/ma19153199

**Published:** 2026-07-27

**Authors:** Shang Wu, Wenxiang Wu, Zhiyang Chen, Liang Tang, Feng Pan

**Affiliations:** 1 School of Materials Science and Engineering, Tsinghua University, Beijing 100084, China; samuel@cnzztech.net (S.W.); panf@mail.tsinghua.edu.cn (F.P.); 2 Zhejiang Zhongze Precision Technology Co., Ltd., Jiaxing 314500, China; chenzy@cnzztech.net (Z.C.); tangl@cnzztech.net (L.T.)

**Keywords:** AA8014 aluminum alloy, burst vents, notch, second-phase particles, recrystallization, burst pressure

## Abstract

**Highlights:**

- The Avrami exponent *n* = 1.87 and activation energy *Q* = 156 kJ/mol are first quantified for recrystallization in the notched AA8014 alloy.
- A critical temperature window of 300–350 °C is identified, above which recrystallization and burst pressure become insensitive to process variations.
- A linear model relating the recrystallized fraction to the burst pressure is established, with prediction errors less than 4.1%.

**Abstract:**

In this study, isothermal annealing of AA8014 aluminum alloy burst vents was performed at 250–500 °C for 10 s to 2 h, and Johnson–Mehl–Avrami–Kolmogorov (JMAK) kinetic analysis was employed to determine the recrystallization parameters at 300 °C. An Avrami exponent of *n* = 1.87 and an apparent activation energy of *Q* = 156 kJ/mol were obtained, revealing a recrystallization mechanism driven by high stored energy and synergistically regulated by particle-stimulated nucleation at coarse second-phase particles and Zener pinning by fine Al(Fe,Mn)Si dispersoids. The burst pressure evolution was highly temperature-dependent: annealing at or below 300 °C led to sluggish recrystallization and a gradual pressure decline, whereas annealing at 350 °C and above resulted in recrystallization completion within 10 s and a sharp pressure drop to a stable plateau of approximately 0.92 MPa. The Al(Fe,Mn)Si dispersoids showed no significant differences in size distribution or grain-boundary pinning after 1 h at both 300 °C and 500 °C. This invariance across the tested range rendered the microstructure and burst performance insensitive to process variations. A quantitative predictive model correlating the recrystallized fraction with the burst pressure was established, with prediction errors less than 4.1%. The 300–350 °C interval is identified as the critical temperature window for regulating recrystallization kinetics and burst pressure, providing a rational basis for the heat-treatment design of burst vents.

## 1. Introduction

The rapid growth of electric vehicles and large-scale energy storage has continuously increased the energy density of lithium-ion batteries, making thermal runaway a critical safety bottleneck [1,2]. As the ultimate safety device in a battery system, the burst vent must deliver accurate and consistent burst pressure to ensure reliable protection. In industrial practice, a trapezoidal or V-shaped notch is created on an aluminum disc by precision stamping. This local thinning forms a weak geometric spot, enabling the vent to rupture precisely and release pressure during thermal runaway. This technique is favorable because of its simplicity, cost-effectiveness, and good reproducibility. However, the severe nonuniform deformation introduced by stamping produces a microstructure in the notch region that is markedly different from that of the base material, which complicates the precise control of subsequent heat treatments.

To date, most studies have focused on the macroscopic design of burst vents and their pressure-relief characteristics, examining parameters such as opening pressure, relief area, and vent geometry [3,4]. Some investigations have also explored electrically controlled valves to reduce response delays [5]. Furthermore, the set burst pressure significantly influences the thermal runaway behavior of the cell; for 18650 batteries, both the gas release volume and the vent opening time increase notably when the burst pressure is raised from 1 to 3 MPa [6]. Thus, precise control of the burst pressure is essential not only for valve reliability but also for overall battery safety. Nevertheless, the quantitative influence of annealing on the notch-region microstructure and its consequent effect on burst performance remain poorly understood [7]. A primary reason is the lack of quantitative kinetic data for recrystallization under the unique strain gradient field in the notch, which prevents the establishment of a deterministic process–property link.

AA8014 (Al–Fe–Mn) alloys are widely used for burst vents because of their specific strength, corrosion resistance, and formability. Its microstructure is highly dependent on the thermomechanical history. In the as-cast state, the main phases are α-Al, Al_6_(Fe,Mn), and minor amounts of Al_3_(Fe,Mn) and α-Al(Fe,Mn)Si. During homogenization, metastable Al_6_(Fe,Mn) particles gradually transform into stable α-Al(Fe,Mn)Si with a micro-porous structure. Subsequent rolling and annealing further modify the morphology, size, and distribution of the Fe-bearing phases, producing both micron- and submicron-sized dispersoids [8,9]. These particles regulate recrystallization through the competing mechanisms of particle-stimulated nucleation (PSN) and Zener pinning: coarse particles promote nucleation, whereas fine dispersoids hinder grain-boundary migration. The balance between these two effects governs the recrystallization kinetics and the final grain size [10,11]. During stamping, the notch undergoes much more severe plastic deformation (86% thickness reduction) than the matrix does, resulting in the development of a high-dislocation-density substructure. Consequently, recrystallization in this localized region is the critical link between microstructural evolution and macroscopic burst performance.

The recrystallization of aluminum alloys with uniform microstructures has been extensively studied. Zener pinning has been shown to effectively suppress recrystallization in AA3104 [12] and in AlMgScZr alloys containing Al_3_(Sc,Zr) dispersoids [13]. Zhou et al. systematically investigated the kinetics and texture evolution during the annealing of cryogenic-rolled 3003 alloys [14]. In high-Fe Al–Mg–Si–Cu alloys, transition elements (Mn, Mo, V, and Cr) influence recrystallization by controlling α-Al(Fe,TM)Si dispersoids [15]. Moreover, the efficiency of the PSN is strongly affected by the strain level and the size and distribution of the second-phase particles [16,17].

Unlike these classical cases of uniform deformation, the notch region in the present study features a steep strain gradient and an 86% thickness reduction. The mechanism by which this geometric gradient influences recrystallization nucleation, growth, and the synergistic role of second-phase particles remains poorly understood. The kinetic parameters (the Avrami exponent *n* and the apparent activation energy *Q*) for recrystallization in the notch have not been quantitatively reported, and a quantitative model relating the recrystallized fraction *X* to the burst pressure *P*_burst_ is lacking. Without a clear understanding of the “process–microstructure–performance” relationship, heat-treatment design remains empirical, which severely limits optimization and product consistency.

Hence, systematic isothermal annealing experiments were carried out on pre-notched AA8014 aluminum alloy burst vents over a wide processing window (250–500 °C, 10 s–2 h). The objectives were to quantify the recrystallization kinetics in the notch region, reveal the synergistic action of the second-phase particles during recrystallization, establish a quantitative correlation between the recrystallized fraction and the burst pressure, and identify a robust processing window that ensures both high performance and tolerance to parameter variations. This study aims to provide a theoretical basis for the rational design of heat-treatment procedures for burst vents.

## 2. Materials and Methods

**Figure 1:**
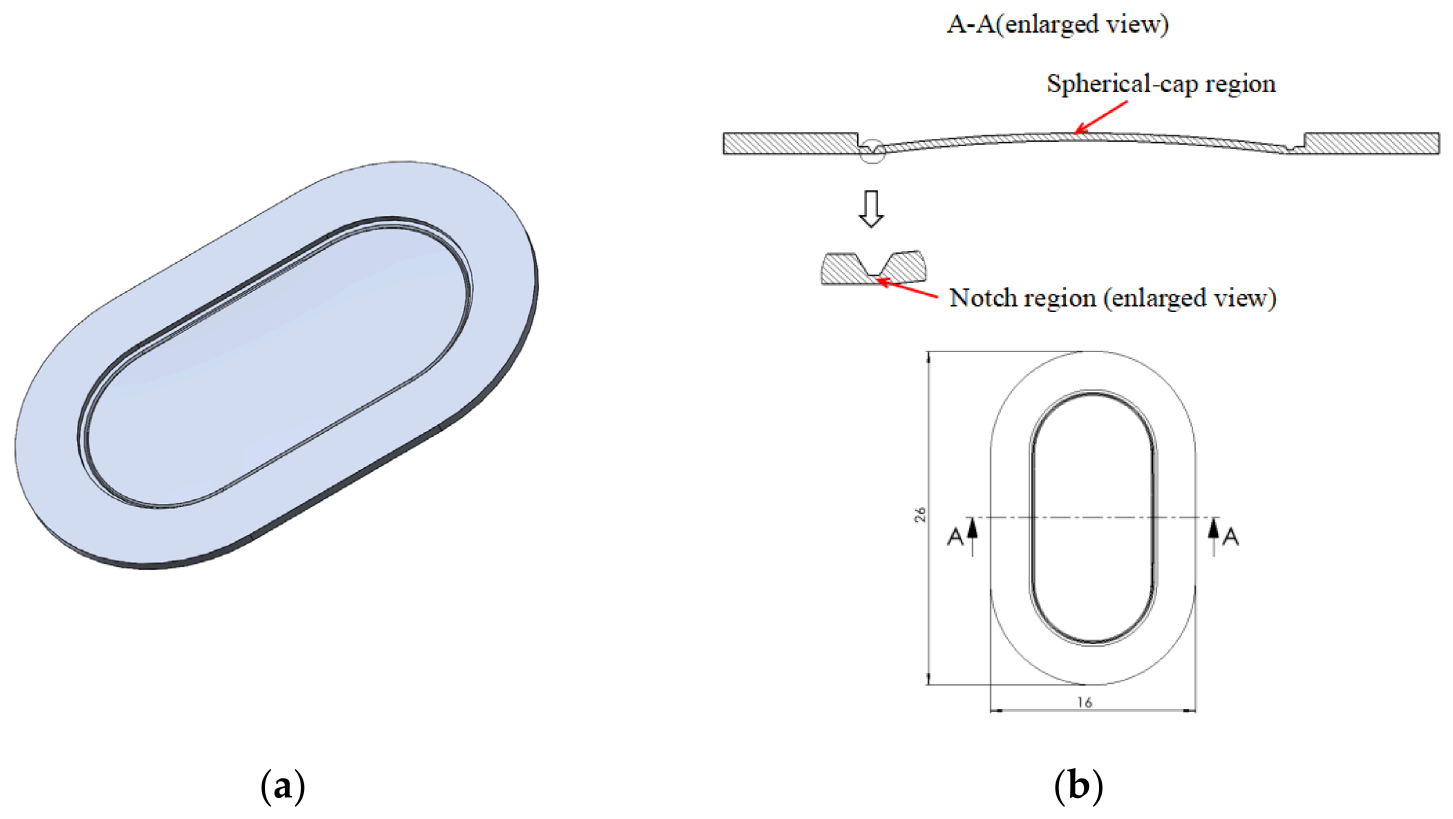

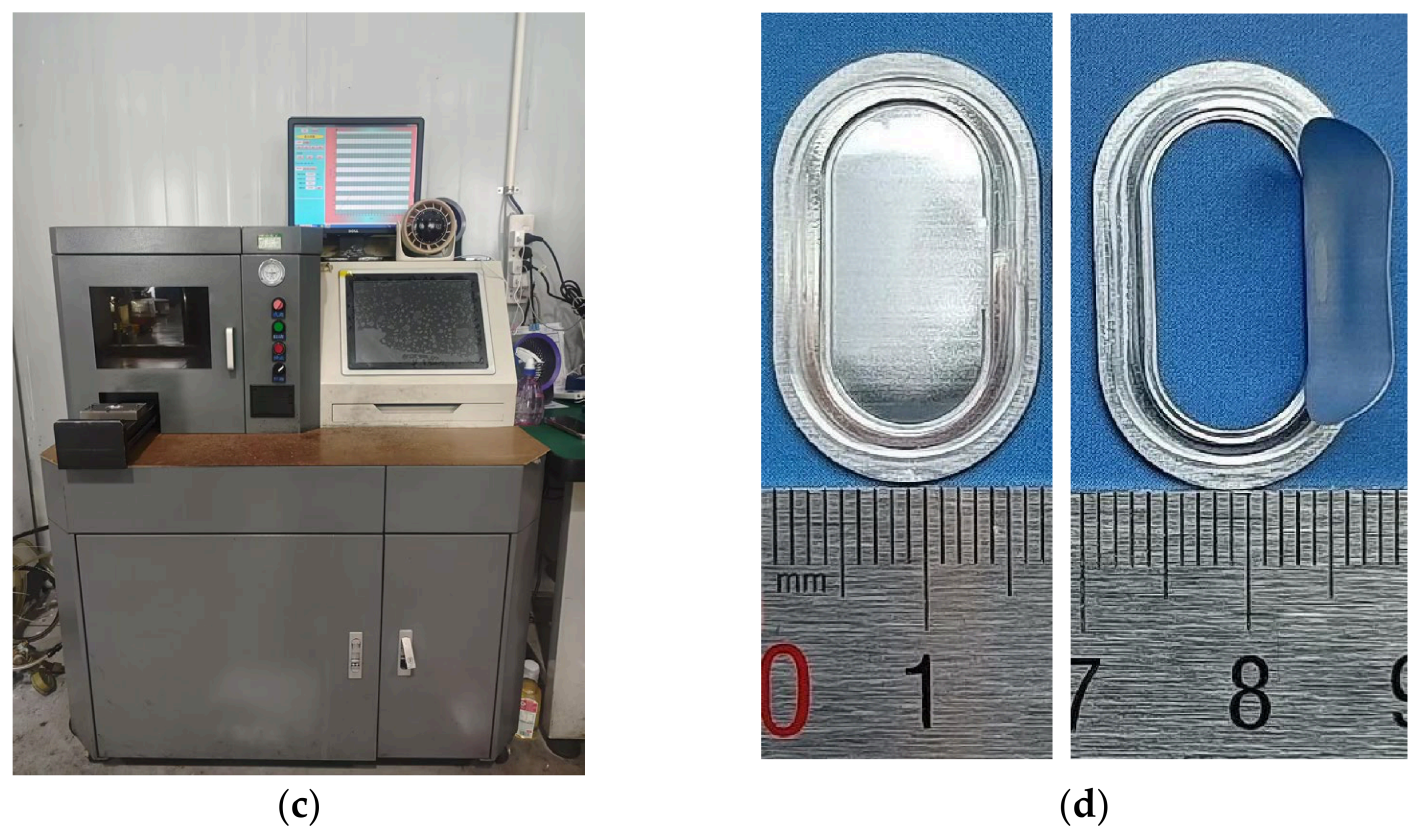
Schematic illustration and photographs of the burst vent and testing setup: (**a**) 3D structure of the vent; (**b**) cross-sectional details of the notch; (**c**) burst pressure testing setup; (**d**) representative burst vents before (**left**) and after (**right**) burst testing.

Commercial O-temper AA8014 aluminum alloy strips with a thickness of 0.5 mm were used as the raw material, and their chemical composition is shown in Table 1. Trapezoidal notched burst vent specimens were fabricated by precision stamping. The three-dimensional structural schematic and cross-sectional geometric details of the vent are displayed in Figure 1a and Figure 1b, respectively. The residual thickness in the notch region was 0.07 mm (86% reduction), whereas the adjacent spherical-cap region had a thickness of 0.18 mm (64% reduction). Such a sharp thickness gradient induces a pronounced stress concentration at the notch, which promotes preferential deformation and eventual fracture under internal pressure, thereby ensuring reliable vent opening at the predetermined design pressure.

Isothermal annealing of the burst vents was carried out in a KSY-30-12 salt-bath furnace (Wuhan Yahua Electric Furnace Co., Ltd., Wuhan, China) at 250, 300, 350, 400, 450, and 500 °C, with holding times of 10 s, 30 s, 60 s, 5 min, 30 min, 1 h, and 2 h, respectively, at each temperature. Samples were directly placed into a furnace preheated to the set temperature and, after annealing, rapidly quenched in water to retain the high-temperature microstructure.

For microstructural characterization, cross-sections of the notch region from both the as-stamped and annealed samples were prepared by standard metallographic procedures, including mechanical grinding, polishing, electropolishing, and anodic coating. An Olympus BX53M (Olympus Corporation, Tokyo, Japan) optical microscope was used to observe the grain morphology, measure the recrystallized grain size, and evaluate the recrystallized fraction. For the recrystallized fraction X, ten measurement lines (spaced 10 μm apart) were placed uniformly across the width of the notch region on each micrograph. The lines ran along the thickness direction and spanned the entire notch cross-section (length ≈ 70 μm). For each line, the total length of segments intercepted by recrystallized grains (L_rex_) was measured and divided by the total line length (L_total_) to obtain a local recrystallized fraction. The average of the ten lines gave the fraction for that field of view, and the average over five parallel samples was taken as the recrystallized fraction X for that annealing condition, with the standard deviation serving as the error bar. For the recrystallized grain size, the linear intercept method was used on micrographs taken from fully recrystallized notch regions. Two measurement lines were placed along the diagonal direction; the number of intersections with grain boundaries was counted for each line, and the average intercept length was converted to the grain size. The average over five parallel samples was reported, again with the standard deviation.

A Sigma 300 field-emission scanning electron microscope (SEM) (Carl Zeiss Microscopy GmbH, Oberkochen, Germany) equipped with an Xplore 30 energy-dispersive spectrometer (EDS) (Oxford Instruments, Abingdon, UK) was used to examine the morphology and composition of the second-phase particles and to observe the burst fracture surfaces.

Transmission electron microscopy (TEM) specimens were mechanically thinned to less than 0.1 mm. At this stage, the notch region (original thickness of 0.07 mm) was visibly thinner than the surrounding area, so its location could be precisely identified. A 3-mm-diameter disc was punched out with the notch at its center and further thinned to less than 50 μm, followed by argon ion milling. Owing to thickness variations, the notch zone undergoes preferential thinning during ion milling, which spontaneously forms an electron-transparent region localized at the score line. Observations were made on a JEOL JEM-F200 transmission electron microscope (JEOL Ltd., Tokyo, Japan) operated at 200 kV. Bright-field imaging revealed the morphology and distribution of the second-phase particles, and the attached EDS system was used for mapping and point analysis to determine their elemental composition. For statistical analysis of particle size, multiple bright-field TEM images were taken from different areas of the notch region for each annealing condition. More than 120 particles with a size smaller than 1 μm were manually measured. For spherical particles, the diameter was recorded; for elongated particles, the maximum length was measured. The average particle size and size distribution were then calculated.

Burst pressure measurements were conducted using a BP-HP-02 high-precision tester (Shenzhen Jiaruipu Technology Co., Ltd., Shenzhen, China). The vent was sealed in the fixture, after which high-pressure nitrogen was introduced at a constant rate until rupture, and the instantaneous burst pressure was recorded. The testing setup is shown in Figure 1c. Representative vents before and after burst testing are shown in Figure 1d. For statistical reliability, five samples were tested under each condition, and the average value was calculated.

The accuracy and precision of the measurement equipment are as follows. The BP-HP-02 burst pressure tester has a specified accuracy of 0.005 MPa according to the manufacturer’s technical specifications. The ZEISS Sigma 300 field-emission SEM provides a secondary electron resolution of ≤1.0 nm at 15 kV, which is sufficient for characterizing the micron-scale second-phase particles and fracture surfaces. The JEOL JEM-F200 TEM offers a point resolution of ≤0.23 nm at 200 kV, enabling clear observation of dispersoid morphology and grain-boundary pinning. The Olympus BX53M optical microscope has a resolution of approximately 0.4 μm, which is adequate for measuring the recrystallized grain size (8–10 μm).

## 3. Results

### 3.1. Microstructural Evolution

**Figure 2:**
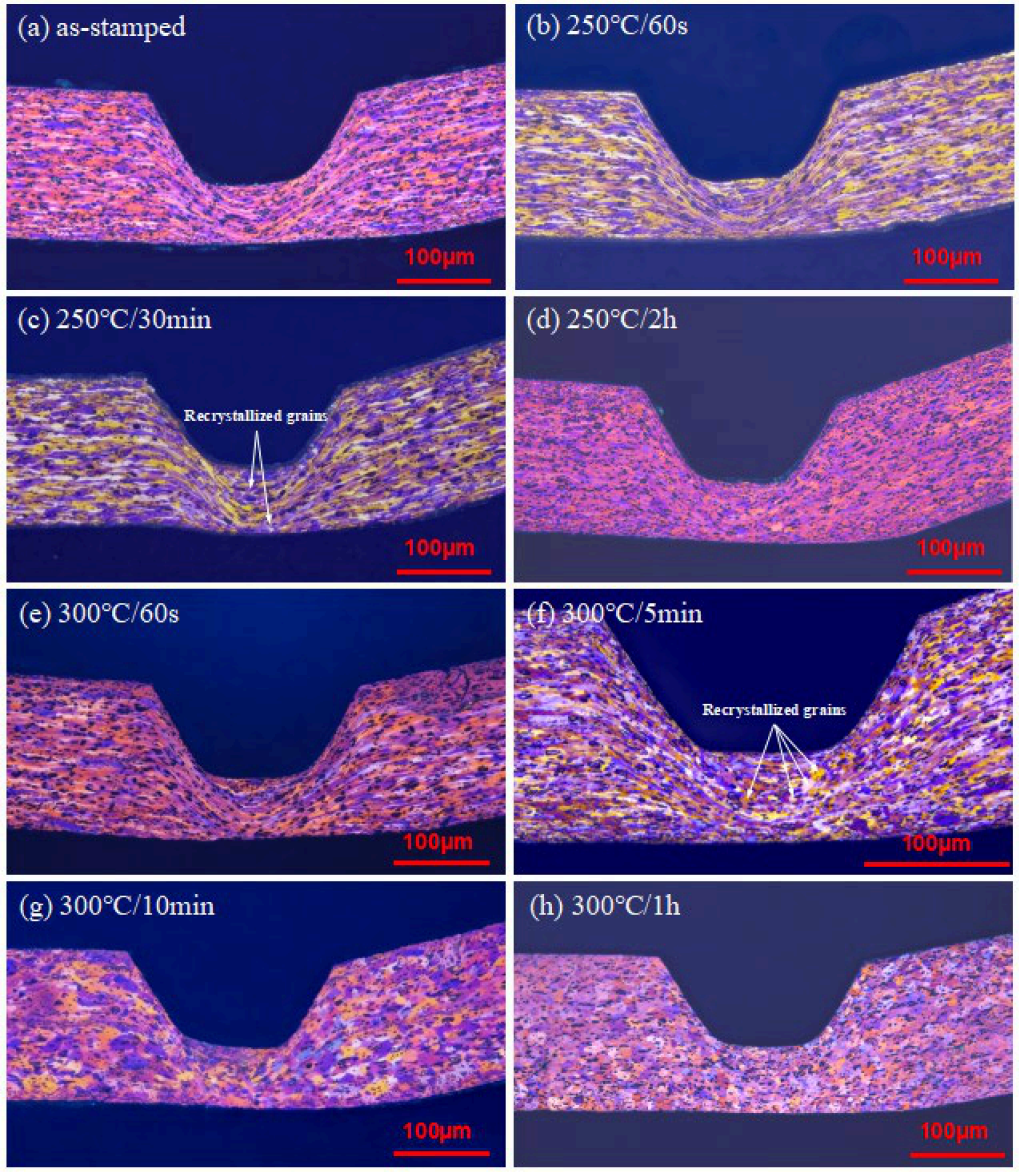
Microstructural evolution in the notch region after annealing at 250 °C and 300 °C for different durations: (**a**) as-stamped; (**b**) 250 °C/60 s; (**c**) 250 °C/30 min; (**d**) 250 °C/2 h; (**e**) 300 °C/60 s; (**f**) 300 °C /5 min; (**g**) 300 °C/10 min; (**h**) 300 °C/1 h. The colors arise from anodic coating under polarized light.

The microstructural evolution in the notch region strongly depended on the annealing temperature and time. The as-stamped sample showed a typical fibrous structure as a result of severe plastic deformation (Figure 2a). At 250 °C, recrystallization was extremely slow: no obvious change was observed after 60 s (Figure 2b); after 30 min, a few fine equiaxed grains appeared only in the notch region, where the stored energy was highest, while the spherical-cap region remained fibrous, showing a clear “selective recrystallization” feature (Figure 2c); even after 2 h, the notch region was not fully recrystallized (Figure 2d), indicating insufficient atomic diffusion at this temperature. At 300 °C, the process showed a distinct time dependence. During the first 60 s, the structure remained in the recovery stage (Figure 2e). After 5 min, partial recrystallization began in the notch (Figure 2f), and after 10 min, recrystallization was complete (Figure 2g). Extending the holding time to 1 h did not cause any significant grain coarsening (Figure 2h).

**Figure 3:**
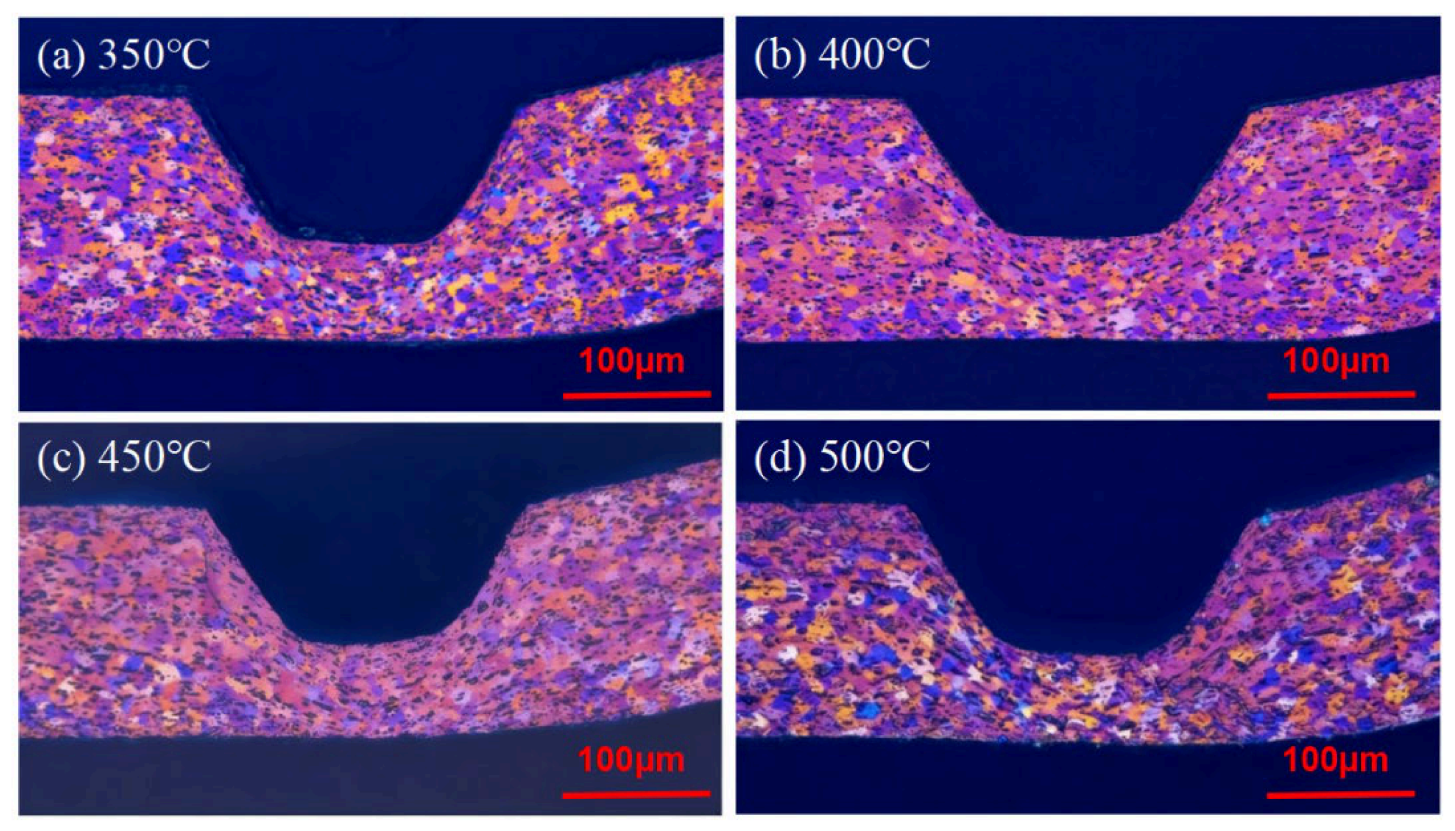
Microstructure of the notch region after annealing at different temperatures for 10 s: (**a**) 350 °C; (**b**) 400 °C; (**c**) 450 °C; (**d**) 500 °C. The colors arise from anodic coating under polarized light.

**Figure 4:**
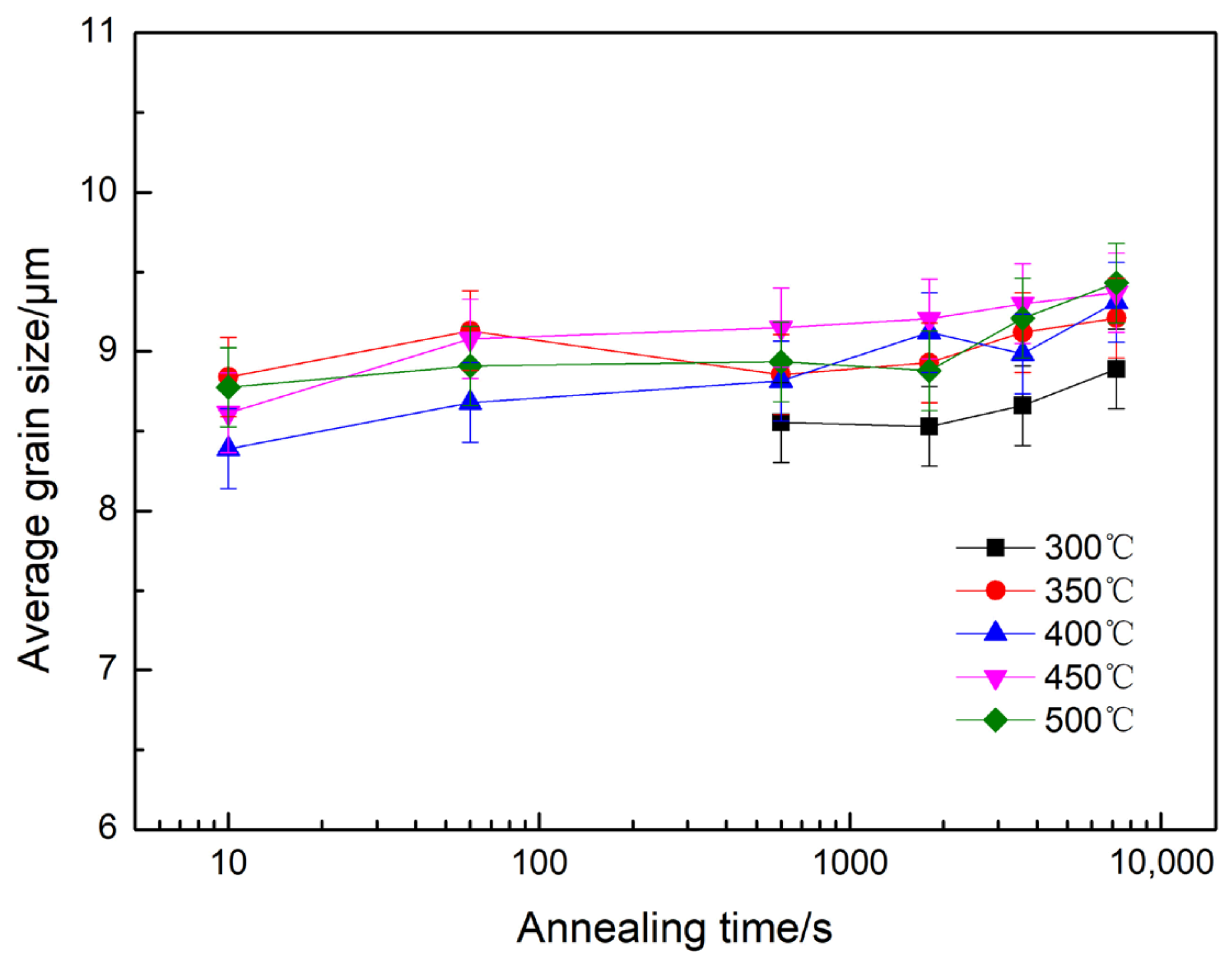
Grain size evolution in the notch region with annealing time at different temperatures.

A critical transition in recrystallization behavior occurred at annealing temperatures of 350 °C and above. In the range of 350–500 °C, the notch region achieves complete recrystallization within 10 s, generating a uniform and fine equiaxed grain structure (Figure 3). The average grain size exhibits negligible temperature dependence within this temperature range. The evolution of grain size with respect to temperature and time is shown in Figure 4. At 300 °C, after recrystallization was complete (e.g., at 10 min and 1 h), the grain size stabilized at 8–10 μm. Over the broad window of 350–500 °C and 10 s–2 h, the recrystallized grain size also remained consistent in the 8–10 μm range, demonstrating microstructural stability within the tested range. In addition, owing to the higher stored energy providing more nucleation sites, the recrystallized grains in the notch were slightly finer than those in the adjacent spherical-cap region.

### 3.2. Stability of Second-Phase Particles

**Figure 5:**
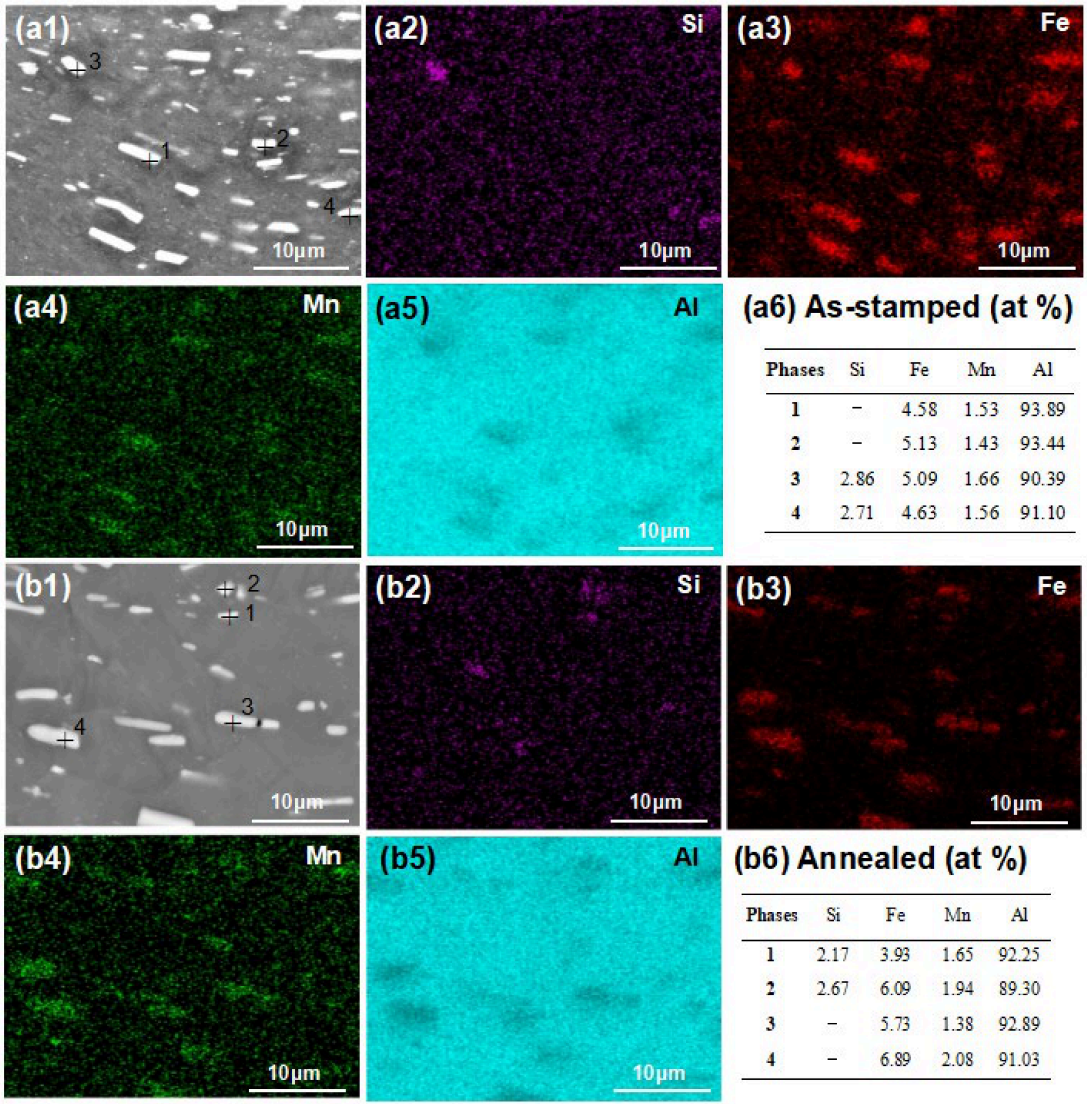
SEM images and EDS elemental maps of coarse second-phase particles in the notch region: (**a1**–**a6**) as-stamped; (**b1**–**b6**) after 500 °C/2 h of annealing.

**Figure 6:**
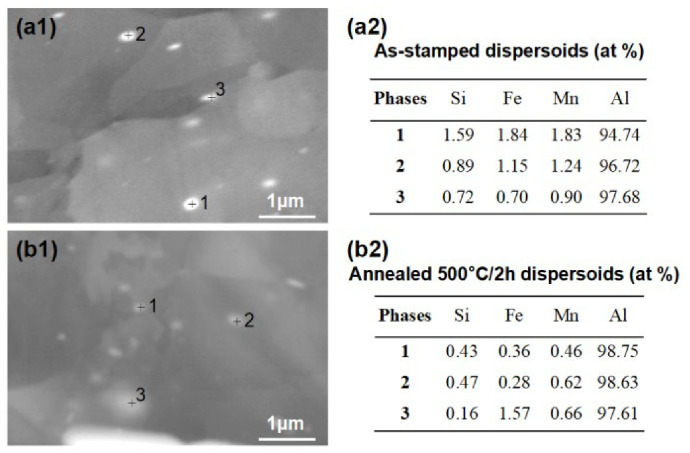
High-magnification SEM images and EDS analyses of fine dispersoids: (**a1**,**a2**) as-stamped; (**b1**,**b2**) after 500 °C/2 h of annealing.

Two types of second-phase particles were present in the as-stamped notch region. The first type was coarse particles larger than 1 μm; their morphology and elemental maps are shown in Figure 5(a1–a5), with the results of the EDS point analyses given in Figure 5(a6). The second type consisted of fine particles smaller than 1 μm that were uniformly dispersed in the matrix; their high-magnification images and EDS results are shown in Figure 6(a1) and Figure 6(a2), respectively. EDS indicated that most coarse particles were rich in Fe and Mn, while a few also contained Si. The fine dispersoids consistently contained Fe, Mn, and Si. In agreement with previous work on similar alloys [9], these particles are identified as Al-(Fe,Mn) and Al-(Fe,Mn)-Si intermetallic compounds.

To verify the thermal stability of these particles, the as-stamped and 500 °C/2 h annealed samples were first compared by SEM. As shown in Figure 5(b1–b6) and Figure 6(b1,b2), after 500 °C/2 h of annealing, the morphology, type, size, and distribution of both the coarse and fine particles did not change significantly; no obvious coarsening or dissolution occurred, indicating excellent inherent thermal stability.

**Figure 7:**
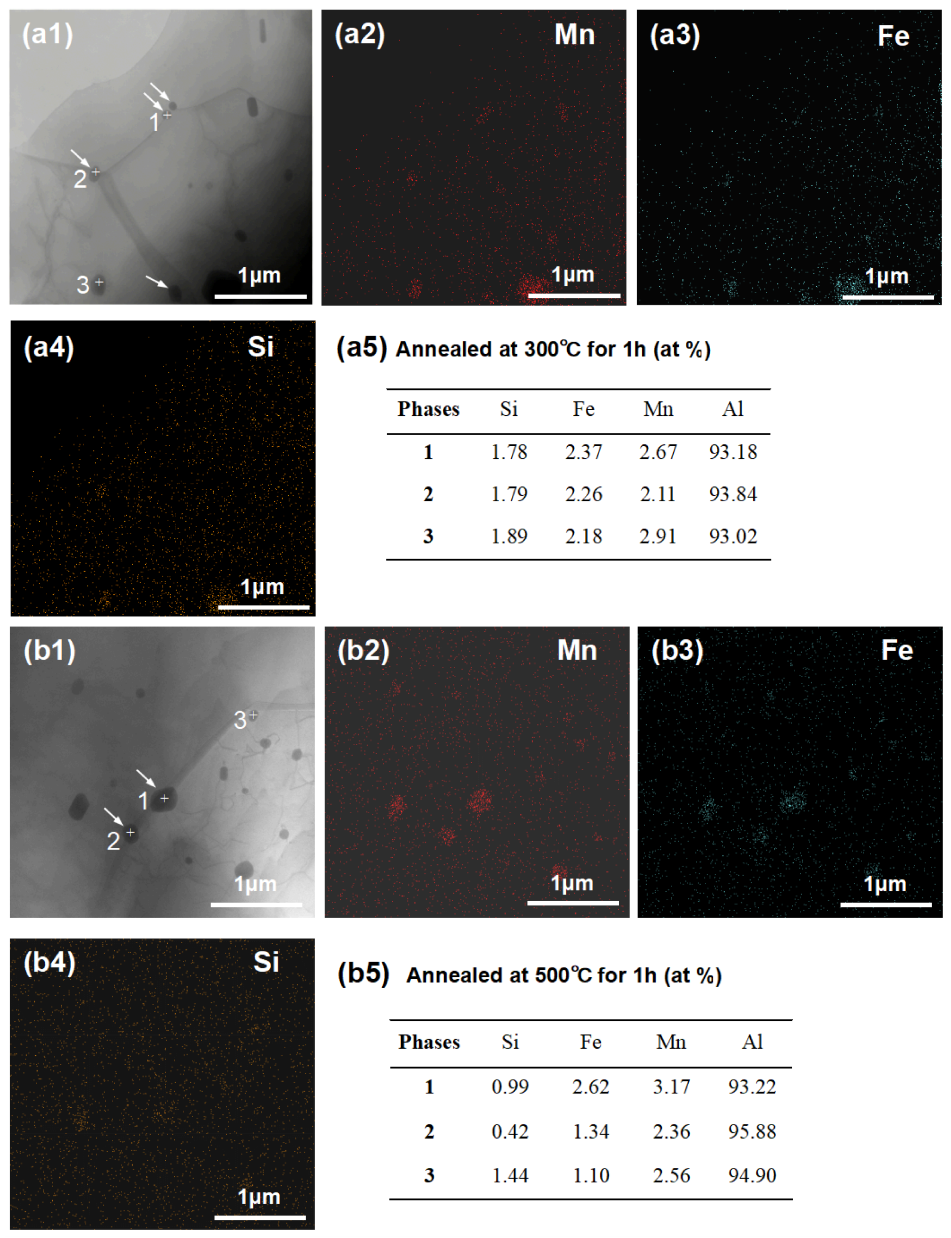
TEM analysis of dispersoids after annealing at 300 °C/1 h and 500 °C/1 h: (**a1**) bright-field image for 300 °C/1 h, showing pinning on grain boundaries (white arrows); (**a2**–**a4**) corresponding EDS maps (Mn, Fe, Si); (**a5**) EDS point analysis of a typical particle; (**b1**) bright-field image for 500 °C/1 h; (**b2**–**b4**) elemental maps; (**b5**) EDS point analysis.

To further investigate the stability of the dispersoids’ pinning effect and explain the stable grain size across the 300–500 °C range, TEM analysis was performed on samples annealed at 300 °C/1 h and 500 °C/1 h. Figure 7(a1,b1) shows that in both states, the dispersoids were fine and uniformly distributed, with some particles located on the grain boundaries (white arrows). EDS mapping and point analysis (Figure 7(a5,b5)) confirmed that these particles are enriched in Mn, Fe, and Si. No significant differences in morphology, size, distribution, grain-boundary pinning, or composition were observed between the two states. These findings demonstrate that the dispersoids remain stable within the 300–500 °C range and that their pinning effect does not weaken at higher temperatures, providing a direct microstructural explanation for the stable grain size.

**Figure 8:**
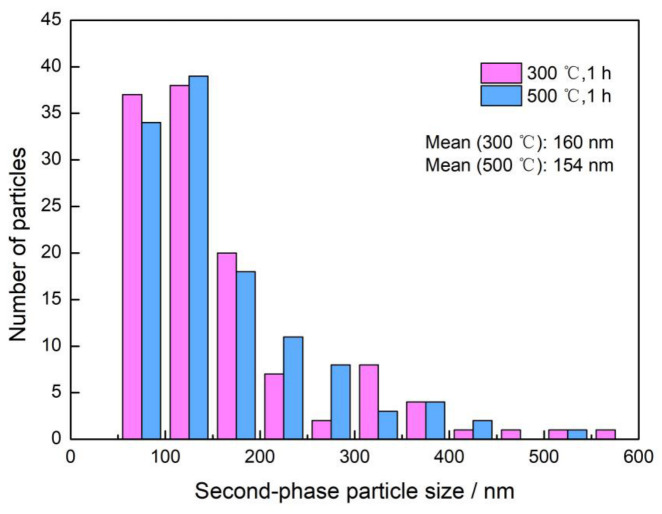
Size distribution histograms of second-phase particles in the notch region after annealing at 300 °C/1 h and 500 °C/1 h.

Figure 8 presents the size distributions of second-phase particles (<1 μm) in the notch after 1 h of annealing at 300 °C and 500 °C. For each condition, more than 120 particles were measured from multiple TEM micrographs. The distributions exhibit a dominant peak in the 50–150 nm range, with progressively fewer particles in the 150–350 nm range, and only a minor fraction exceeding 350 nm under either condition. The mean particle sizes are 160 nm at 300 °C and 154 nm at 500 °C, yielding a negligible difference of only 6 nm. Over 60% of the particles fall within the 50–150 nm interval under both conditions. These quantitative results confirm the excellent thermal stability of the dispersoids against coarsening across the examined temperature range.

### 3.3. Evolution of the Burst Pressure

**Figure 9:**
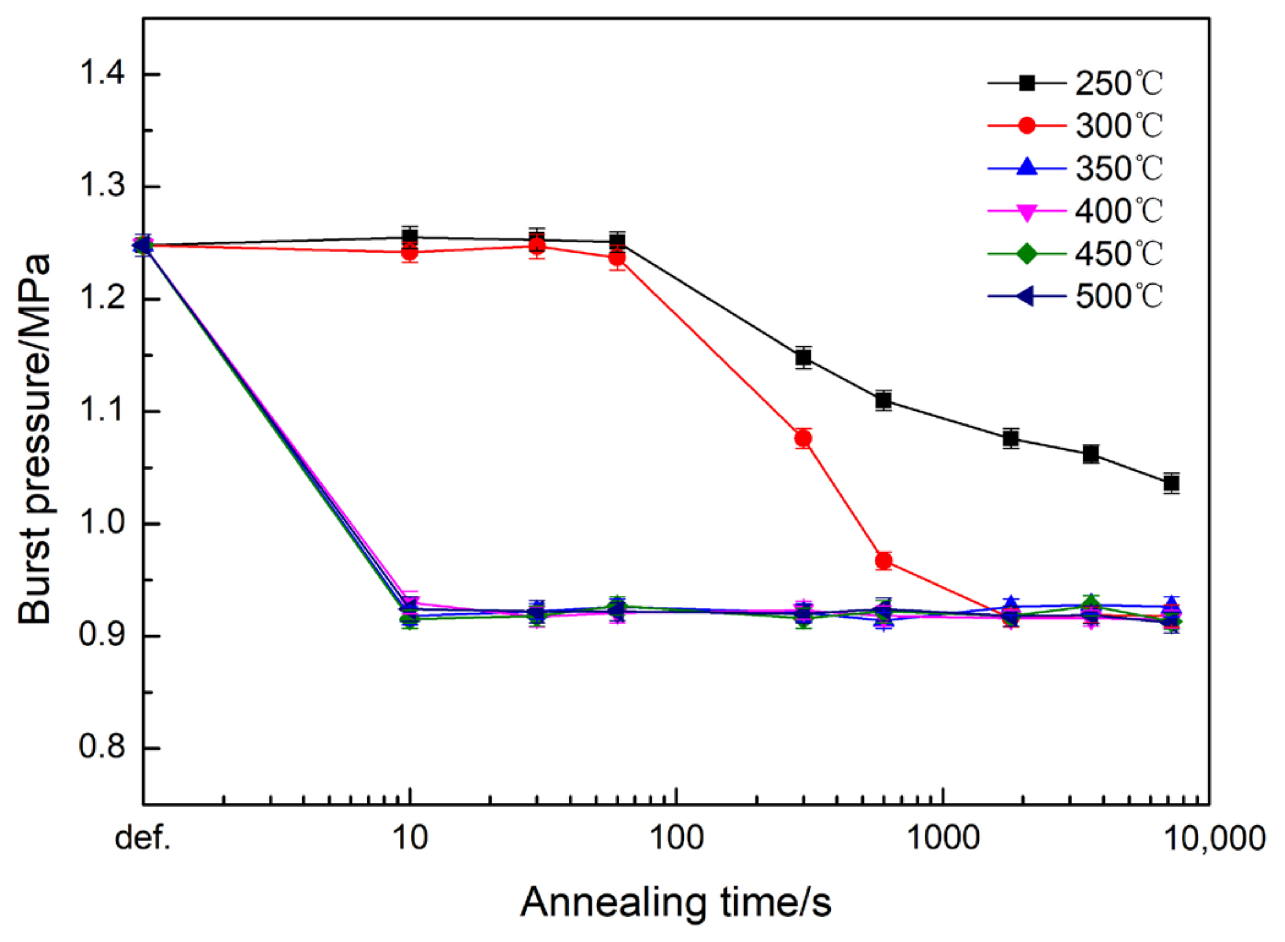
Burst pressure as a function of annealing temperature and time.

The burst pressure as a function of the annealing conditions exhibited three characteristic regimes (Figure 9). At 250 °C, the burst pressure decreased slowly and continuously with holding time; it had not stabilized even after 2 h, corresponding to incomplete recrystallization. At 300 °C, the burst pressure remained unchanged during the initial 60 s, then decreased sharply with progressing recrystallization, and eventually stabilized at ~0.92 MPa after 30 min—a duration that coincided with the completion of recrystallization. In the 350–500 °C range, at all temperatures, the burst pressure sharply decreased to the same stable plateau value (≈0.92 MPa) immediately after 10 s of annealing. Extending the holding time to 2 h caused no further change, indicating strong process robustness within the parameter window examined.

**Figure 10:**
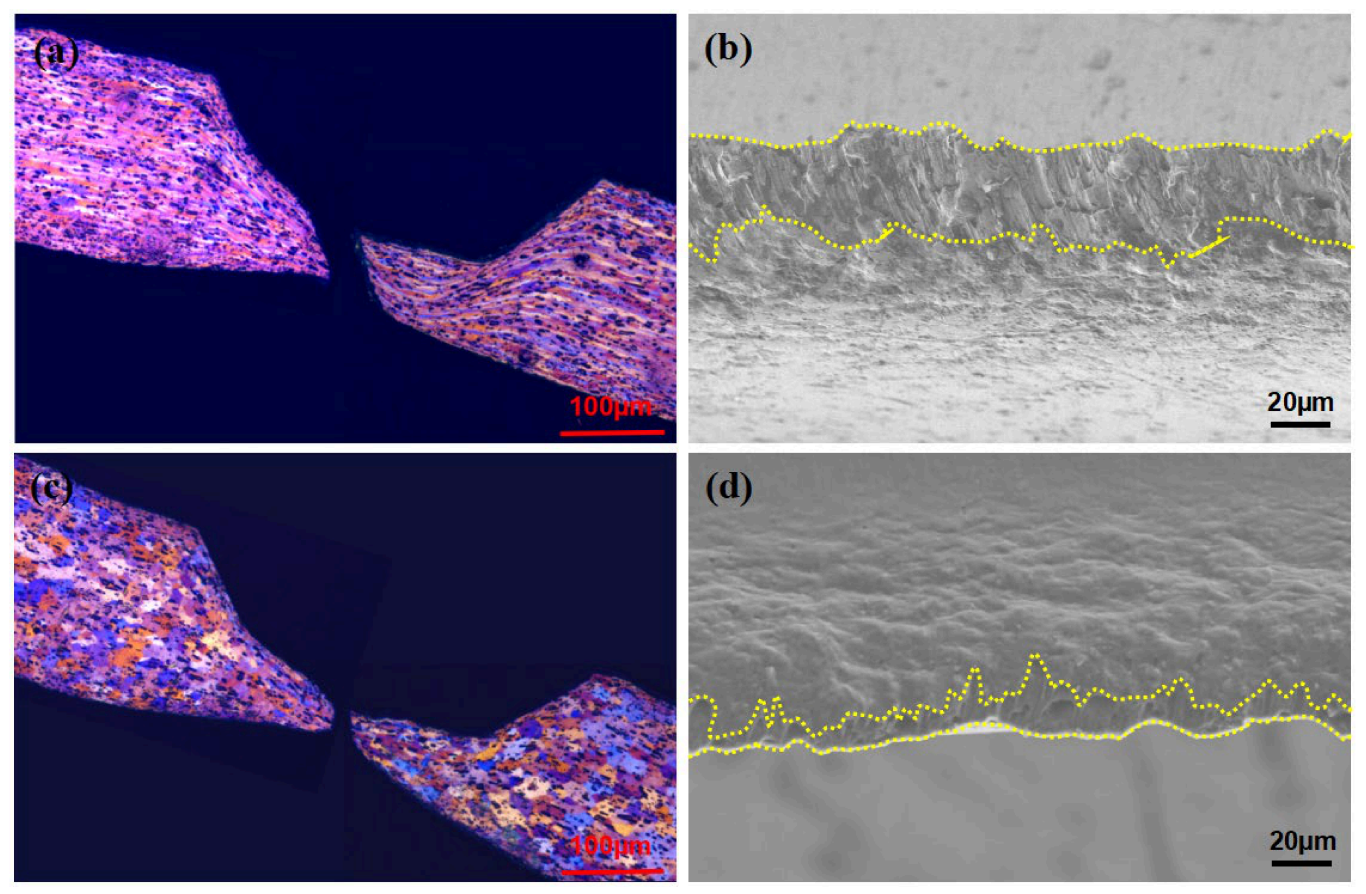
Fracture features of the notch region after burst testing: (**a**,**b**) cross-sectional metallograph and SEM fractograph of the as-stamped sample; (**c**,**d**) those of the 300 °C/1 h recrystallized sample. The colors in the metallographs (**a**,**c**) arise from anodic coating under polarized light. The dotted lines in (**b**,**d**) mark the boundaries of the fracture regions observed in the SEM images.

The fracture characteristics after burst testing are shown in Figure 10. The cross-section of the as-stamped sample shows that the fracture path is inclined at approximately 45° to the principal stress direction (Figure 10a), and the SEM fractograph reveals elongated shear dimples of uneven size and shallow depth (Figure 10b). This suggests that the high-dislocation-density fibrous structure limited uniform plastic deformation and that fracture was dominated by shear. In contrast, the fully recrystallized sample (300 °C/1 h) exhibited clear necking (Figure 10c), and the SEM fractograph showed deeper, more uniform equiaxed dimples (Figure 10d). The fine equiaxed grain structure enhanced uniform plastic deformation, and the fracture mode changed to microvoid coalescence, providing a microscopic explanation for the stabilization of the burst pressure after complete recrystallization.

## 4. Discussion

### 4.1. Recrystallization Behavior in the Notch Region

During stamping, the notch region experiences local strains that are far greater than those in the matrix due to the abrupt cross-sectional change. The thickness reduction is as high as 86%, creating a high-dislocation-density fibrous structure with greater stored energy than that in the spherical-cap region. This provides a strong thermodynamic driving force for recrystallization [10], which explains why recrystallization starts preferentially in the notch.

**Figure 11:**
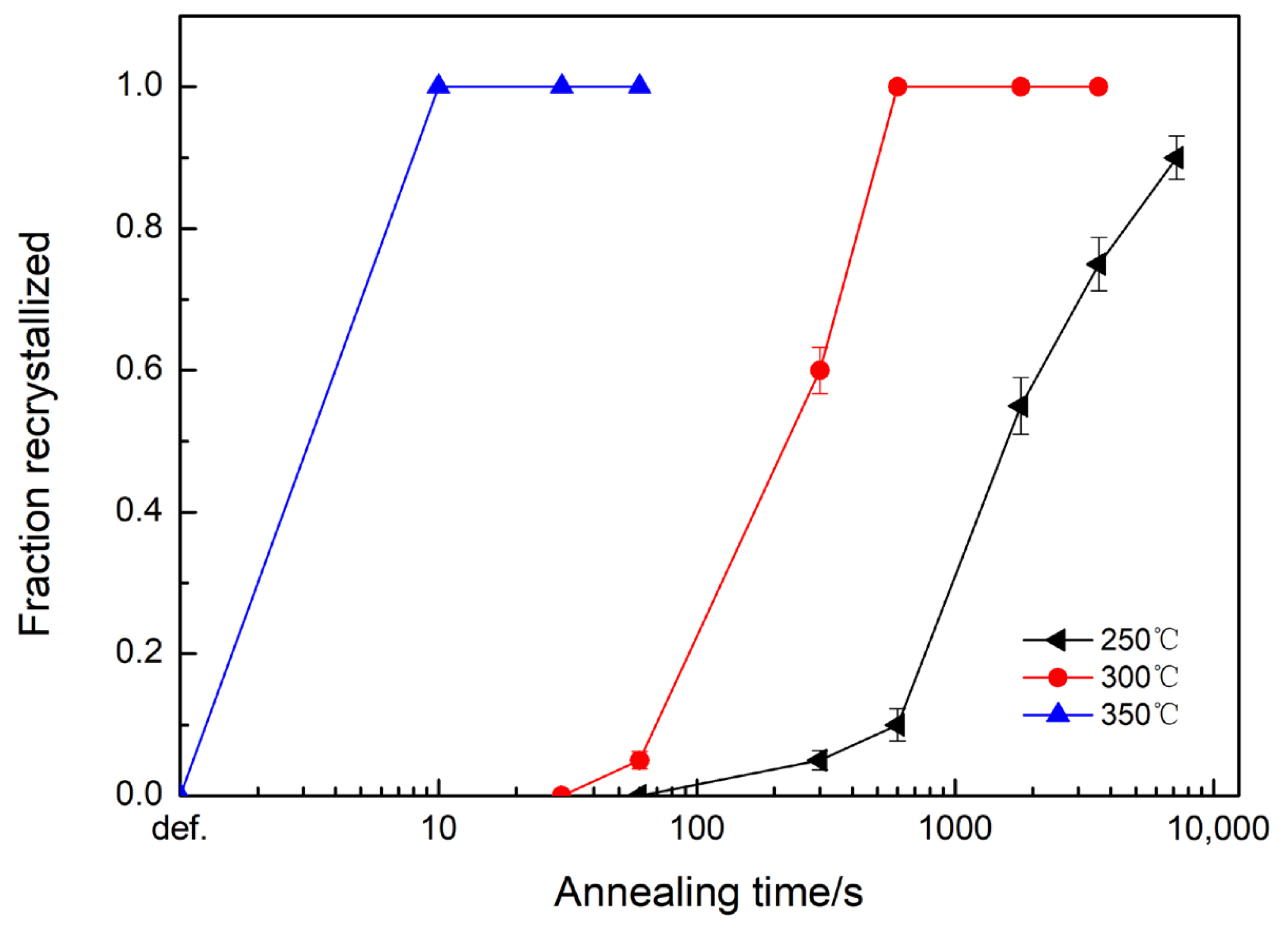
Recrystallized fraction X in the notch region as a function of annealing time at 250 °C, 300 °C, and 350 °C.

The recrystallized fraction X as a function of temperature and time is plotted in Figure 11 (for 400–500 °C, recrystallization was completed within 10 s, similar to 350 °C, so these data are not repeated). The behavior can be divided into three distinct regimes. At 250 °C (sluggish regime), the atomic thermal activation is insufficient; the recrystallization rate is extremely low, and the notch is not fully recrystallized even after 2 h. At 300 °C (time-dependent regime), a classic sigmoidal kinetic curve is observed; X reaches approximately 60% at 5 min and 100% after 10 min. At 350–500 °C (instantaneous regime), recrystallization is completed within 10 s, resulting in the formation of a uniform fine equiaxed structure. This study identifies 300–350 °C as the critical temperature window where recrystallization behavior undergoes a fundamental transition.

The recrystallized grain size remains stable at 8–10 μm in the 350–500 °C range. This stability arises from the synergistic effect of the two types of particles. Coarse Al(Fe,Mn) particles (>1 μm) promote nucleation via PSN, increasing the nucleation rate and refining the grains [18,19]. Fine, dispersed Al(Fe,Mn)Si particles (<1 μm) are uniformly distributed and strongly pin the grain boundaries, thereby suppressing grain coarsening [20]. The combination of a high nucleation rate and strong pinning gives the microstructure remarkable thermal stability.

According to Zener pinning theory, the pinning pressure is proportional to the particle volume fraction and inversely proportional to the particle radius [10]. In the present samples, the dispersoids after 500 °C/1 h annealing are fine (average radius ≈ 77 nm; Figure 8) and uniformly distributed, thereby capable of providing effective pinning. TEM observations (Figure 7) directly confirm that these particles are located on the grain boundaries. This pinning is sufficiently strong to inhibit post-recrystallization grain coarsening, which explains why the grain size remains in the 8–10 μm range across the entire 300–500 °C temperature interval.

TEM observations (Figure 7) directly confirmed that there was no significant difference in the morphology, size, distribution, or grain boundary pinning characteristics of the dispersoids after annealing for 1h at 300 °C and 500 °C. This invariance demonstrates that the dispersoids remain thermally stable across this temperature range, with no weakening of their pinning effect at elevated temperatures—a behavior that underpins the stable grain size, which depends not on the absolute temperature but on the dispersoid stability itself. Furthermore, as shown in Figure 5 and Figure 6, neither the coarse second-phase particles nor the dispersoids underwent coarsening or dissolution after annealing at 500 °C for 2 h. This stability arises from the inherent thermal stability of the Al(Fe,Mn)Si phase (which exhibits only limited coarsening at 500 °C [21]) and the low diffusivity of Fe and Mn in the Al matrix [22,23]. Given that the applied annealing temperatures (≤500 °C) are considerably lower than the critical temperature (≥610 °C) for the phase transformation from Al(Fe,Mn) to Al(Fe,Mn)Si [8], the second-phase particles are confirmed to maintain a thermodynamically stable state throughout the heat treatment process.

To quantitatively characterize the recrystallization kinetics of the notch region, the Johnson–Mehl–Avrami–Kolmogorov (JMAK) equation was applied to fit the isothermal annealing data at 300 °C:

*X* = 1 − exp(−*kt^n^*) (1)

where *X* is the recrystallized fraction, *t* is the holding time, *k* is the rate constant, and *n* is the Avrami exponent. Taking the double logarithm of both sides yields:

ln[−ln(1 − *X*)] = ln*k* + *n*ln*t* (2)

**Figure 12:**
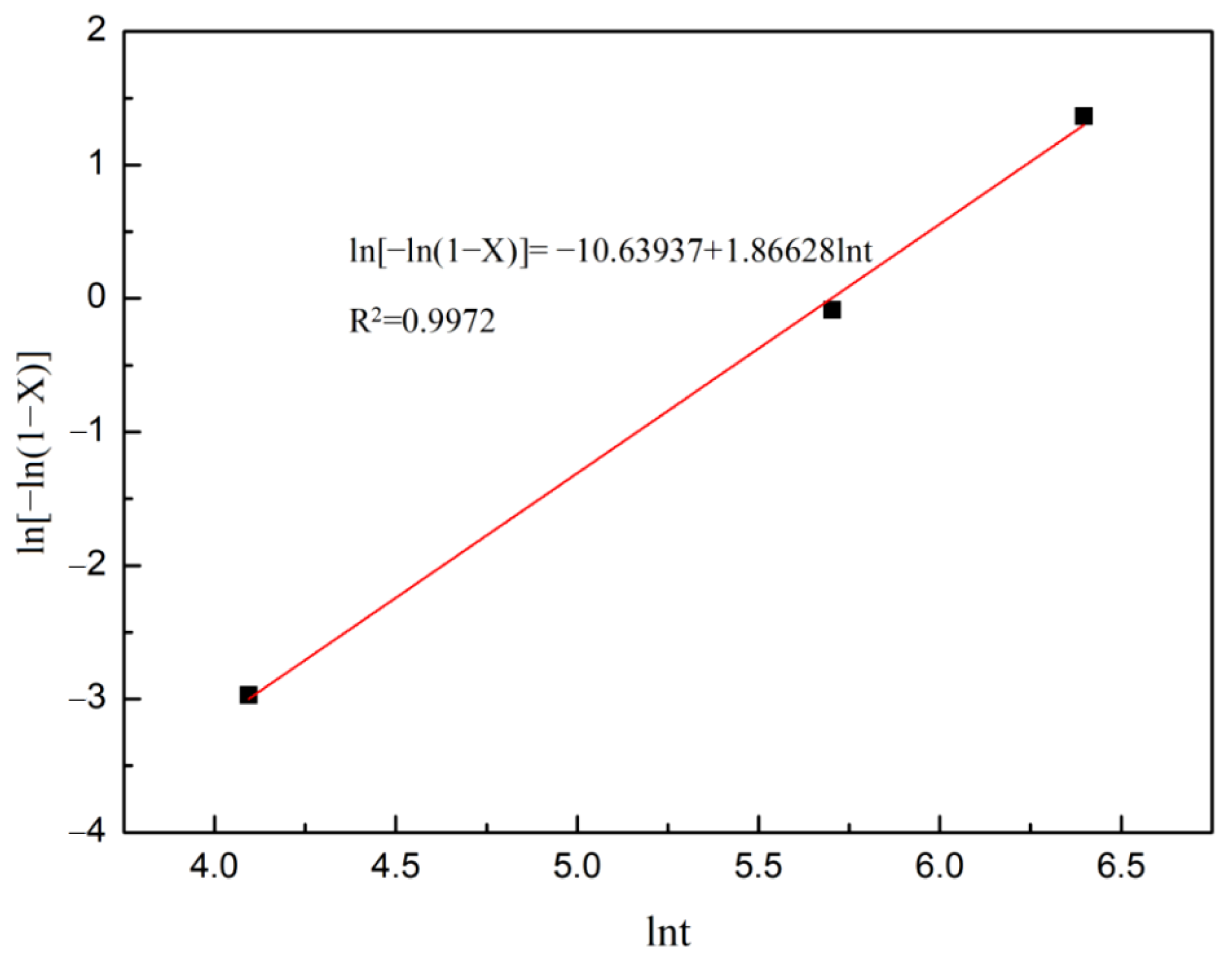
JMAK plot of ln[−ln(1 − X)] versus lnt for isothermal annealing at 300 °C. Black squares denote experimental data, and the red solid line represents the linear fit of the JMAK equation.

The fitting yielded *n* ≈ 1.87 (Figure 12) with an R^2^ of 0.9972, indicating an excellent fit. This *n* value, which is close to the theoretical value of 2, suggests that recrystallization in the notch region is dominated by pre-existing nucleation sites (site saturation) [10]. An *n* close to 2 means that the nucleation rate decays rapidly to near zero at the very beginning of annealing, which is entirely consistent with the high stored energy and high strain gradient in the notch. High stored energy facilitates extensive nucleation in the early stage; subsequently, the increase in the recrystallized fraction is dominated by the growth of these nuclei within the deformed matrix. The Zener pinning effect suppresses grain coarsening after recrystallization, causing *n* to be slightly less than 2.

**Figure 13:**
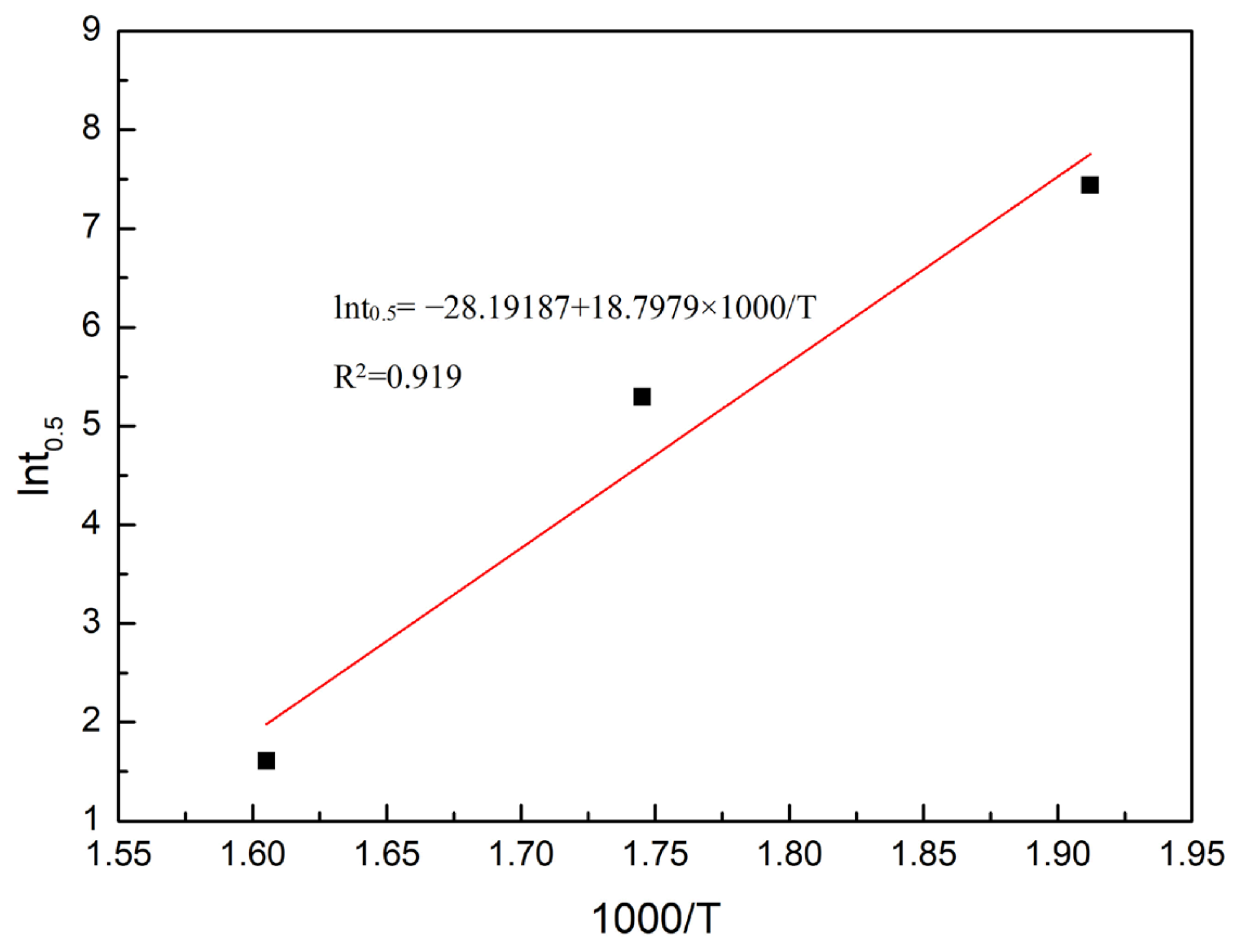
Linear relationship between ln *t*_0.5_ and 1000/*T*. Black squares denote experimental data, and the red solid line represents the linear fitting.

On the basis of the time required to reach the 50% recrystallized fraction, t_0.5_, at different temperatures, the apparent activation energy for recrystallization, Q, was calculated using the Arrhenius equation [10]:

*t*_0.5_ = *C*·exp(*Q*/*RT*) (3)

where *C* is a constant, *Q* is the activation energy for recrystallization (kJ/mol), *R* is the gas constant, and *T* is the absolute annealing temperature (K). The *t*_0.5_ values corresponding to 250 °C, 300 °C, and 350 °C were 1700 s, 200 s, and 5 s, respectively. From the linear fit of ln *t*_0.5_ versus 1000/*T* (Figure 13), the apparent activation energy was determined to be *Q* = 156 kJ/mol. This value is higher than the activation energy for recrystallization in pure aluminum (≈126 kJ/mol) [24], indicating that the pinning effect of the second-phase particles adds significant resistance. The extra ~30 kJ/mol can be quantitatively attributed to the Zener drag exerted by the fine dispersoids on grain-boundary migration. This provides experimental evidence for quantifying the drag effect. Moreover, the PSN from coarse particles and the high stored energy in the notch compensate for this resistance, preventing the overall activation energy from rising even further.

### 4.2. Microstructure–Burst Pressure Correlation and Robust Processing Window

#### 4.2.1. Microstructural Dependence of the Burst Pressure

**Figure 14:**
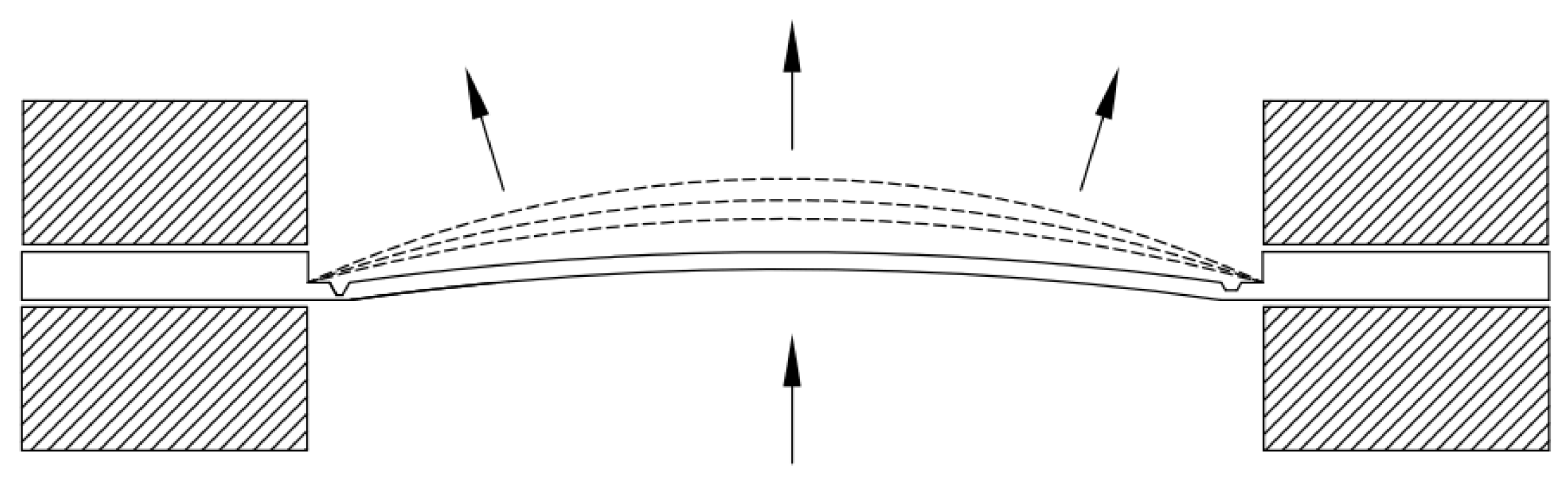
Schematic of burst vent bulging deformation under internal pressure. The lower vertical arrow denotes the direction of internal pressure, and all upper arrows represent the outward arching displacement of the burst vent.

The burst vent can be simplified as a peripherally clamped elliptical thin shell (Figure 14). Under internal pressure, the center bulges axisymmetrically, and the disc experiences biaxial tensile stresses. The notch, where the thickness decreases from 0.18 mm to 0.07 mm, acts as a stress concentration, and bursting initiates there.

For a burst vent with a fixed geometry, the burst pressure *P*_burst_ is proportional to the fracture strength of the notch region material, *R*_m_, and the residual thickness, *t*, as follows:

*P*_burst_ ∝ (*R*_m_·*t*)/*K* (4)

where *K* is a geometric constant. Thus, for a given geometry, *P*_burst_ scales linearly with *R*_m_, which in turn depends on the microstructural state. Wang et al. [25] investigated flat-scored metal diaphragms and identified diaphragm thickness and notch depth as the key geometric parameters, whereas material tensile strength constitutes the fundamental mechanical factor. Their findings are fully consistent with the proportionality expressed in Equation (4).

In the as-stamped condition, the severe plastic deformation introduced a high dislocation density and substantial work hardening in the notch region. This raised the fracture strength *R*_m_ to its peak value, yielding the highest burst pressure of 1.25 MPa. During annealing, recrystallization softens the material by reducing the dislocation density, leading to a decrease in *R*_m_. The degree of softening can be quantitatively characterized by the recrystallized fraction *X* (0 ≤ *X* ≤ 1). Since *P*_burst_ is proportional to *R*_m_, *P*_burst_ can be expressed as a function of *X*:

*P*_burst_ = *P*_rex_ + (*P*_def_ − *P*_rex_)·(1 − *X*) (5)

where *P*_def_ is the burst pressure in the as-stamped state and *P*_rex_ is that after full recrystallization. This equation quantitatively correlates macroscopic performance with the microstructural variable *X*. The model assumes that the mechanical responses of recrystallized and non-recrystallized domains obey the simple rule of superposition under isostrain loading, which constitutes a reasonable first-order engineering approximation.

#### 4.2.2. Quantitative Link Between Recrystallization Kinetics and Burst Pressure Evolution

On the basis of the recrystallization kinetic model in Section 4.1, for annealing at 300 °C, the JMAK equation is as follows:

*X*_300_(*t*) = 1 − exp(−*kt^n^*), *k* = e^−10.639^, *n* = 1.87 (6)

**Figure 15:**
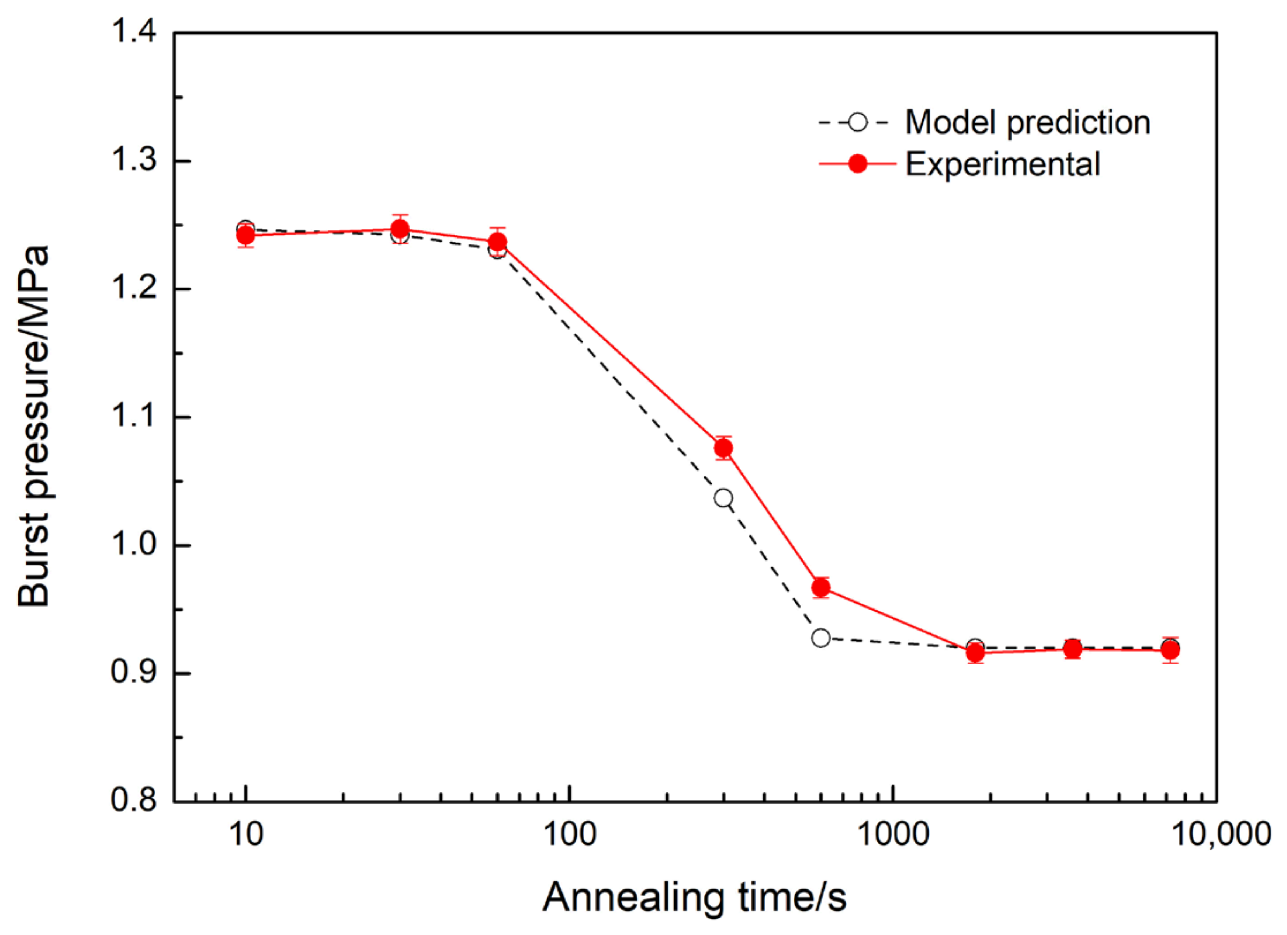
Comparison of experimental burst pressure values with model predictions for annealing at 300 °C.

Substituting Equation (6) into Equation (5) allows the burst pressure evolution at 300 °C to be predicted. As shown in Figure 15, the model predictions (black dashed line) agree well with the experimental data (red solid line). The relative deviations range from 0.11% to 4.07%, averaging 1.21%. These results validate the model and, more importantly, confirm that the recrystallized fraction *X* is the key intrinsic parameter governing the burst pressure. This enables burst performance to be inferred from microstructural measurements. Possible sources of deviation include uncertainties in the JMAK fitting, statistical errors in the metallographic quantification of *X*, and experimental scatter in the burst pressure measurements (standard deviation ≈ ±0.01 MPa for five samples). For process design purposes, the prediction accuracy is acceptable. In the high-temperature range (350–500 °C), recrystallization is complete within 10 s (*X* = 1); Equation (5) therefore directly gives *P*_burst_ = *P*_rex_, accounting for both the instantaneous drop and the subsequent stable plateau.

Notably, Equation (5) is based on the simplified assumption that, for a fixed notch geometry, *X* is the dominant factor. In reality, burst pressure may also be affected by recovery, local strain distribution, and residual stresses. The validity of the model is strictly limited to the specific geometry and alloy studied. For different notch depths, angles, or other alloys, the values of *P*_def_ and *P*_rex_ and their relationships to *X* may require recalibration. The extension of this study to other systems should be performed with caution, and the results should be validated with independent data.

Based on the kinetic analysis, two distinct regimes of burst pressure response emerge. In the nonrobust window (250–300 °C), atomic diffusion is sluggish, recrystallization is governed by kinetic constraints, and *X* increases slowly with time, rendering the burst pressure highly sensitive to holding time; consequently, process fluctuations may introduce appreciable scatter in performance. In the robust window (350–500 °C), recrystallization completes within 10 s. The dispersoids remain stable and their pinning effect sustains with temperature, attesting to the excellent thermal stability of the fully recrystallized microstructure. Consequently, the burst pressure is insensitive to variations in both annealing temperature and holding time. Macroscopic performance thus stems from intrinsic material properties—namely dispersoid pinning—rather than from precise control of processing parameters. The robust window identified here highlights the intrinsic link between recrystallization, microstructural stability, and process tolerance.

## 5. Conclusions

The recrystallization kinetics and burst pressure response of pre-notched AA8014 aluminum alloy burst vents were systematically investigated through isothermal annealing over a wide temperature (250–500 °C) and time (10 s to 2 h) range, using OM, SEM, TEM, and burst pressure testing. The main conclusions are as follows:

(1) Quantitative kinetic analysis of recrystallization in the notch region yields an Avrami exponent of *n* = 1.87 and an apparent activation energy of *Q* = 156 kJ/mol. The *n* value, close to 2, confirms site-saturation nucleation under the high strain gradient. The activation energy, approximately 30 kJ/mol greater than that of pure aluminum, quantitatively reflects the Zener drag exerted by the fine Al(Fe,Mn)Si dispersoids. These findings reveal a recrystallization mechanism driven by high stored energy and synergistically regulated by particle-stimulated nucleation (PSN) at coarse second-phase particles and Zener pinning by fine dispersoids.
(2) The 300–350 °C interval is identified as the critical temperature window where recrystallization changes from time-dependent to instantaneous completion. In the 350–500 °C range, the thermal stability of the Al(Fe,Mn)Si dispersoids is excellent. After 500 °C/1 h of annealing, the average dispersoid size is approximately 154 nm, with most particles in the 50–150 nm range—essentially the same as after 300 °C/1 h of annealing. No significant coarsening or dissolution occurs. Consequently, both the recrystallized grain size (8–10 μm) and the burst pressure (stable at ≈0.92 MPa) are insensitive to process variations, demonstrating outstanding process robustness.
(3) A quantitative model correlating the recrystallized fraction *X* to the burst pressure *P*_burst_ was established as: *P*_burst_ = *P*_rex_ + (*P*_def_ − *P*_rex_)·(1 − *X*). The model predictions agree with the experimental values to within 4.1%, constituting a practical theoretical tool for precise burst pressure control.

The present framework is potentially extendable to other alloy systems and notch geometries. Future work could examine the recrystallization kinetics and burst pressure response in other aluminum alloys with different second-phase characteristics, as well as in alternative materials such as copper and stainless steel. In addition, the coupling between the local strain gradient distribution and recrystallization kinetics warrants further investigation, combining finite element simulation with phase-field or cellular automaton modeling to provide a more mechanistic understanding of nucleation and growth under steep strain gradients. Such efforts would provide more refined theoretical support for the integrated control of microstructure and performance in geometrically gradient materials.

## Figures and Tables

**Table 1:** Chemical composition of the AA8014 aluminum alloy (wt.%).

Fe	Mn	Si	Cu	Mg	Zn	Ti	Al
1.23	0.52	0.07	0.005	0.008	0.006	0.01	Bal.

## Data Availability

The original contributions presented in this study are included in the article. Further inquiries can be directed to the corresponding author.
